# On the lifetime of bioinformatics web services

**DOI:** 10.1093/nar/gkaa1125

**Published:** 2020-12-03

**Authors:** Fabian Kern, Tobias Fehlmann, Andreas Keller

**Affiliations:** Chair for Clinical Bioinformatics, Saarland University, Saarbrücken 66123, Germany; Center for Bioinformatics, Saarland Informatics Campus, Saarbrücken 66123, Germany; Chair for Clinical Bioinformatics, Saarland University, Saarbrücken 66123, Germany; Center for Bioinformatics, Saarland Informatics Campus, Saarbrücken 66123, Germany; Chair for Clinical Bioinformatics, Saarland University, Saarbrücken 66123, Germany; Center for Bioinformatics, Saarland Informatics Campus, Saarbrücken 66123, Germany; Department of Neurology and Neurological Sciences, Stanford University, Palo Alto, CA 94305, USA

## Abstract

Web services are used through all disciplines in life sciences and the online landscape is growing by hundreds of novel servers annually. However, availability varies, and maintenance practices are largely inconsistent. We screened the availability of 2396 web tools published during the past 10 years. All servers were accessed over 133 days and 318 668 index files were stored in a local database. The number of accessible tools almost linearly increases in time with highest availability for 2019 and 2020 (∼90%) and lowest for tools published in 2010 (∼50%). In a 133-day test frame, 31% of tools were always working, 48.4% occasionally and 20.6% never. Consecutive downtimes were typically below 5 days with a median of 1 day, and unevenly distributed over the weekdays. A rescue experiment on 47 tools that were published from 2019 onwards but never accessible showed that 51.1% of the tools could be restored in due time. We found a positive association between the number of citations and the probability of a web server being reachable. We then determined common challenges and formulated categorical recommendations for researchers planning to develop web-based resources. As implication of our study, we propose to develop a repository for automatic API testing and sustainability indexing.

## INTRODUCTION

Scientific web servers and web services are frequently developed to make complex algorithms available to a broad research and user community. They have facilitated substantial contributions to the development of the current research landscape in the life sciences and biomedicine. As one example, the web service to the basic local alignment search tool BLAST, originally published by Altschul in 1990 (1) has become one of the most popular web-based tools in sequence analysis. Also, extensions for protein alignment such as Gapped BLAST and PSI-blast (2) have been made available as web services and are accessed thousands of times each day. Belonging to one of the most frequently applied tools, it is evident that the web service is carefully maintained and continuously improved (3). Similarly, other successful web services such as STRING are regularly updated and maintained (4–6). Also, the European Bioinformatics Institute (EMBL-EBI) provides access to several essential analysis tools via web services that are regularly updated, extended and maintained in a sustainable manner (7).

Web services have become of such a high relevance that the journal Nucleic Acids Research (NAR) dedicates a whole special issue each year to this topic. Staring in 2003 with 131 of the most widely perceived web services from the years before (8), the annual web server issue has steadily extended its scope and has become a world-renowned resource for peer-reviewed web servers (9–11). The most recent web server issue got as much as 269 proposals of which 79 manuscripts were finally accepted after peer-review, resulting in an overall acceptance rate of 29% (12). These numbers underline the tremendous popularity but show that scientific rigorousness must be assured for online implementations as well. It became a perception that computational biology resources lack persistence and usability (13,14). Following the study by Veretnik *et al.* (13) in 2008 that investigated the availability of all NAR web servers published in the preceding 4 years, Schultheiss *et al.* presented a similar but extended analysis in 2011 (15). They found that of the 927 web servers published in NAR between 2003 and 2009, 72% were still available at their original addresses while 9% were gone offline. The study by Schultheiss *et al.* excels by a survey among all authors and a functionality test of each server providing example data. In 2017, the survey by Wren *et al.* (16) highlighted that ∼27% of URLs from web servers decayed since their original publication and that this is a relatively stable phenomena observed among scientific web tools.

Since these studies had been conducted another 1026 web instances have been published in a total of 11 issues in NAR. Here, we set to provide an up-to-date and comprehensive evaluation of the general availability of web services. Thus, we collected 2727 articles describing 2396 unique tools published by PubMed indexed journals from 2010 onwards and tested their availability over time.

The main goals of the present study are: i) to present a comprehensive analysis of the accessibility of web services; ii) to get insights into the availability dynamics of web services over a longer period of time, e.g. to understand differences across weekdays and to estimate the extent of typical downtimes; iii) to evaluate with an experiment whether and to which extent recent web services can be rescued by contacting the corresponding authors; iv) provide an analysis on the dependency between tool metadata such as service hoster or host country and site availability and v) use the observations to formulate reasons for the observed web server decay. We use this information to derive practical recommendations for web server developers to improve upon security, maintainability and user experience, that should ultimately extend the expected lifetime of web services.

## MATERIALS AND METHODS

### Literature search

To get a list of web servers we performed a comprehensive PubMed query using the following search term:

(((http://[title/abstract]) or (www.[title/abstract]) or (https://[title/abstract])) and (‘web server’[title/abstract] or (web service[title/abstract]) or webserver[title/abstract] or ‘web service’[title/abstract] or web-server[title/abstract] or web-service[title/abstract])) and ((‘2010/01/01’[Date–Publication]: ‘3000’[Date–Publication])).

The output resulted in 2727 articles. For each article, the abstract and available meta information was downloaded as CSV file and further processed. From the CSV files we extracted the primary web addresses.

### Filtering of tools

The list of tools was further processed and filtered. In 2327 cases a single Uniform Resource Identifier (URL) was provided whereas in the remaining cases several URLs could be determined. This includes special cases where different web servers have been included or where a mirror URL to the same endpoint has been provided. Other examples for articles with two URLs comprise a direct link to the tutorial or to related databases. In these cases only the URL to the actual tool was selected. Another 12 tools were removed since they rather described meta analyses instead of web services in the common sense as used in this work. In addition, 37 other tools were removed because the mentioned web servers were to be deployed locally or not originally published in the linked articles. Finally, one tool was excluded since it had been retracted in the meantime (17). As a next step, we curated redirects and iteratively removed duplicated tools.

### Download of landing pages

We accessed the web pages by using the *download.file* function of R with the curl method selected. With the parameter -m 30 we restricted the maximum operation time to 30 s and with the -L option up to 50 redirects were allowed.

### Filtering of non-working tools

To classify web pages either in reachable or offline we extended the search beyond the typical error messages (e.g. response codes 403, 404, 406, 502 and 503). Screening manually through the non-accessible web pages we identified 44 phrases such as ‘Maintenance in progress’, ‘has been discontinued’ or ‘Our server is down temporarily’. If one of the determined keywords or phrases could be detected the site was classified as non-accessible. On three non-consecutive days, the number of available tools dropped considerably (to less than 80% compared to the preceding day), potentially to technical issues at national hub nodes or internet service providers. Therefore, the affected daily counts were excluded from downstream analysis.

### Additional curation steps for static analysis

After the collection of the long-term availability statistics for all tools we semi-automatically curated the entries by inspecting the URLs provided by *bio.tools* (18), searching for patterns in failed URLs and manually checking for new URLs of unavailable services. In addition, we improved the PubMed provided URLs linking to lab homepages using the address to the corresponding tool explicitly. Furthermore, we also curated tool URLs that are reachable but host different and irrelevant content. Tools that were affected by these steps were then excluded from the long-term analysis. After this final step, a total of 2396 of 2727 tools remained (Supplementary Table S1). Notably, in this set also databases with a limited web service functionality were kept. For the tools that were available but modified their host URL without a suitable scientific publication we provide separate statistics in Supplementary Table S2. Downstream analyses have been carried out using the binary matrix of *p* = 2396 tools (rows) and *n* = 133 days (columns).

### Determining hosting providers

We first collected the IP addresses of all tools and retrieved usage information, hosting domain and ISP information from two IP information services, IP2Location.com and IPinfo.io. We then manually checked all non-educational and non-governmental entries for cloud hosting providers. When no IP address could be found no hosting provider was derived.

### Determining institutional e-mail addresses

First, e-mail addresses of the corresponding authors were extracted from Web of Science. We then searched the hosting domains in a list of free e-mail provider domains found at https://gist.github.com/okutbay/5b4974b70673dfdcc21c517632c1f984.

### Statistical analyses

All analyses have been carried out with R 3.3.2 GUI 1.68 Mavericks build (7288). To evaluate the availability of tools over time, splines from the *smooth.spline* function with 10 degrees of freedom (DF) were used. Pie charts, ridgeline, violin and bar plots were compiled using ggplot2. Clustered heat maps were generated using the *superheat* function in the superheat package. Hypothesis tests (Student's *t*-test, Wilcoxon rank-sum test) were conducted using the R stats implementations.

## RESULTS

### Study set-up to answer the four research questions

**Figure 1.**
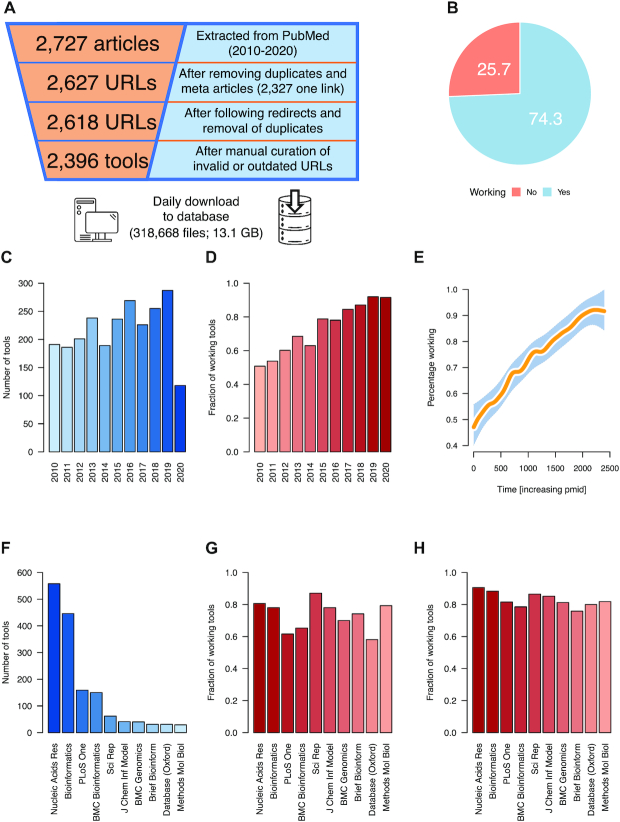
Study set-up and static monitoring. (**A**) Schematic representation of the conducted tool filtering steps. (**B**) Pie chart representing the number of tools that are accessible and not accessible at the start of the observation period. (**C**) Bar chart of the number of tools published by year included in our study. (**D**) Bar chart of the fraction of available tools (snapshot) per publication year. (**E**) Smoothed spline (solid orange line) with surrounding 95% confidence interval (shaded blue area) for the data presented in panel D. (**F**) Number of tools collected per journal. (**G**) Fraction of available tools per journal. (**H**) Available tools per journal restricted to manuscripts published in the past 5 years (2016–2020).

To reach the main goals set we extracted 6727 articles published after 2010 from PubMed. After removing duplicates and false positive hits of our literature search (e.g. meta analyses of tools) 2618 tools remained. After manually curating these (cf. ‘Materials and Methods’ section), 222 tools were removed, retaining the final set of 2396 articles/tools (Supplementary Table S1). The web addresses of the tools were accessed beginning on the 13 April 2020 for a total of 133 days. During this process 318 668 index pages were downloaded and stored to a local database (Figure 1A).

### Static analysis of web services highlights a half-lifetime of 10 years

On 13 April, we completed the first download of all tool landing pages. As a first result 25.7% of the tools were not reachable opposing the 74.3% that were working on that day (Figure 1B). Tracking the number of tools published by year we see a generally increasing trend from ∼200 tools in 2010 to ∼300 tools in 2019 (Figure 1C). These numbers fit well to the overall growth of scientific literature and knowledge (19). With increasing time after publication, we estimate an almost linear decreasing availability of the web services. While tools published in 2019 and 2020 are available to more than 90%, this rate drops to 50% for tools published in 2010 (Figure 1D and E). Considering the source journals, we observe an uneven distribution. As explained in the introduction, the annual web server issue of NAR has become a pivotal resource in the field. Indeed, with over 550 contributions NAR is the leading journal in this regard. However, also Bioinformatics showed a large number of contributions, most likely driven by the application note manuscript category. Two other journals, PLoS One and BMC Bioinformatics reached almost 200 contributions while the remaining ones were distributed among many other journals (Figure 1F). Interestingly, we observed substantial differences in the availability of web servers depending on the journal they were published in. For example, tools published in NAR, Bioinformatics, Scientific Reports or Methods on Molecular Biology had higher long-term availability rates as compared to PLoS One or BMC Bioinformatics (Figure 1G). To further limit the influence of the time variable on these results we repeated the analysis only for the articles published in the past 5 years. Here, the trend of aforementioned differences diminished but was still noticeable (Figure 1H). This first snapshot analysis on the 2396 tools already provides interesting insights on the average lifetime of bioinformatics web services. It is however fair to speculate that these results are influenced by many factors, e.g. the actual weekday when the tools were accessed or seasonal fluctuations. To limit respective effects, we accessed the tools between 13 April and 31 August 2020.

### Monitoring over time indicates short downtimes and higher availability toward the mid of the week

**Figure 2.**
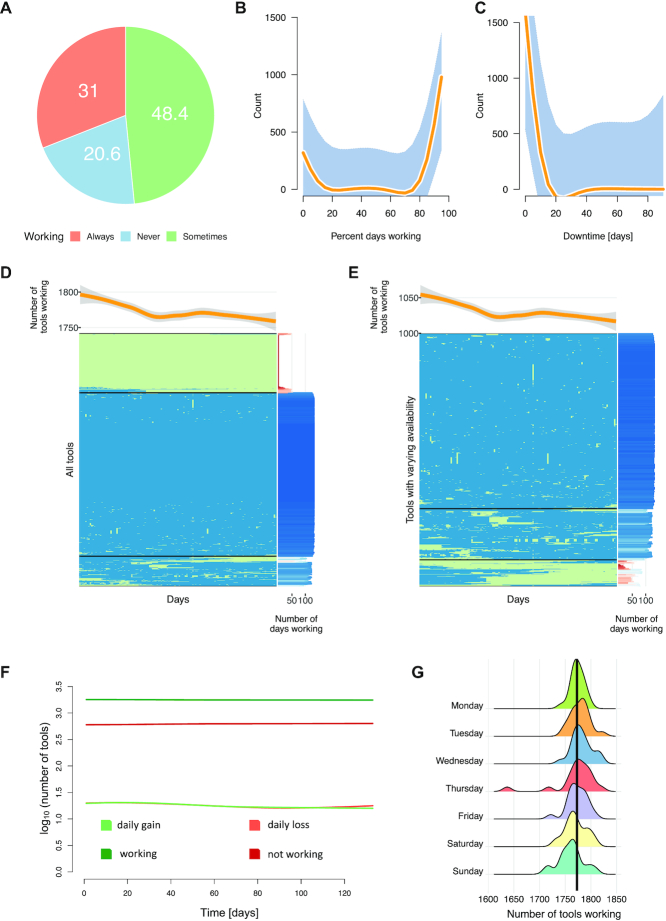
Dynamic monitoring over time. (**A**) Pie chart showing the distribution of tools that were tracked over time into the categories *never available*, *always available* and *sometimes available*. (**B**) Smoothed spline representation of the availability of web servers in percent of days. (**C**) Smoothed spline representation of the observed web server downtime intervals. (**D**) Heat map of the availability matrix for all tools included in the dynamic study. Blue means available, light green not available. The curve on the top represents the smoothed spline representation of the tool availability over time. The histogram on the right shows a bar for each tool proportional to the number of days it was accessible. (**E**) Clustering of those tools that belong to the category of being sometimes available. Notably, this largely corresponds to the middle cluster of panel D but includes also several tools from the other clusters. (**F**) Line chart on the availability categories of tools tracked over time and the trend of daily changes. Toward the end of the observed period we see the green and orange line (lost versus gained per day) diverging. (**G**) Ridgeline plots on the availability of tools per weekday. The solid black vertical line represents the overall mean of tools available per day.

We have collected reliable data on the availability over time and first asked whether and how reachability varies between the tools. We found 31% could always be reached, 20.6% could never be reached and 48.4% could be reached at least once (Figure 2A). The shape of the density distribution of the percentage of days on which tools were working basically supports the existence of these three groups. Only few tools were working between 25 and 75% of the tested days (Figure 2B). For the fraction of tools that was neither consistently off- nor online, we computed the duration of consecutive downtimes. The distribution highlights that individual service outage times were rather short with the computed median and mean downtime of 1 and 2.9 days, respectively (Figure 2C). A clustering of the tools times days availability matrix confirmed the observations on the general functionality of tools (Figure 2D and E). The heat map indicates three main clusters, one with the tools that work always or almost always, one with the tools that work never or almost never and one smaller cluster in the middle with the tools that show a more heterogenous pattern. On average, 1773 of the 2396 tools (74%) were working per day. The minimal number of 1637 tools was reached on 2 July and the maximal number of 1822 tools on Thursday, 28 April. We observed an almost continuous decrease of web server availability along the test timeframe. Deviations from this expectation could possibly be due to two reasons. First, the primary observation of decreasing tool availability over time has been performed on a 10-year horizon while we tracked only another four months, which still might be too short to observe respective long-term trends. Second, as we describe in the next section, we performed a rescue experiment after 2 weeks, which contributed to a temporarily increased tool availability. If we exclude these cases, we again observe the negative correlation between time since publication and fraction of working tools. In line with these results, we detect similar patterns for daily gains and losses of tools over time, only showing divergence toward the end of the observed period (Figure 2F). As last aspect of the analysis we assessed the dynamics on the distribution across weekdays (Figure 2G). The results suggest a tendency of higher availability of tools toward the mid of the week. On average, the lowest number of tools working was obtained on Sundays (∼1761, 73.5%), while on Wednesdays the highest fraction of tools (∼1781, 74.4%) was available. Although the differences are percentage-wise small, still an average of additional 20 tools were working on Wednesdays as compared to Sundays with the difference being statistically significant (*P* = 0.006501).

### Rescue experiment shows that over 50% of web servers can be brought back to service

**Figure 3.**
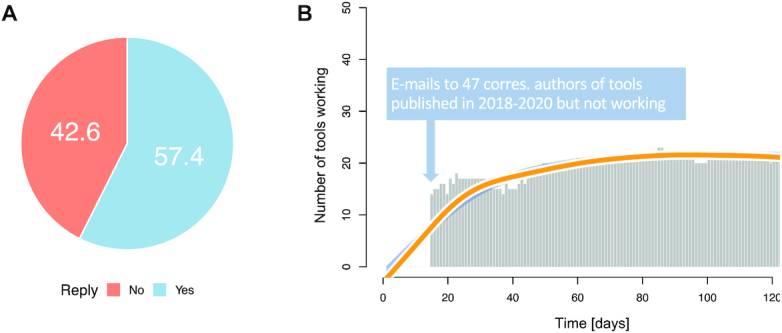
Results of rescue experiment. (**A**) Pie chart of the binarized e-mail responses. (**B**) Bar chart representing the number of tools published in 2019 and 2020 that were not working in the first 2 weeks of the tracking period. The solid orange line represents a smoothed spline with the confidence interval as surrounding blue shaded area.

Our analysis highlights that even tools published in 2019 and 2020 exist that have lost functionality, some even few weeks after their initial publication. Especially in the light of editorial policies requiring the continued availability over at least several years (e.g. the NAR web server issue states: ‘It is expected that the website will be maintained for at least 5 years’) this observation is unexpected. To exclude likely false positives, i.e. tools that were only down for 1 or 2 days because of maintenance work, we compiled a list of tools published in 2019 and 2020 that did not work over the entire first 2 weeks of the observation period. For the resulting 47 instances we contacted the corresponding authors and asked to restore the functionality of the tool. In 57.4% of the cases we got a reply, leaving 42.6% of the enquiries unanswered (Figure 3A). However, the speed of the replies obtained was remarkable: for all but three cases the first reply was received on the same day. The latest reply occurred 3 days after the initial request and altogether, 96 e-mails were exchanged. Already one day after contacting the corresponding authors, 14 tools (29.8%) were brought back to service (Figure 3B). Although this sum slowly increased over the tracking period, we again detected a small decline in availability for the successfully recovered web servers toward the end.

### Frequently cited web services invalidate URLs from scientific publications

**Figure 4.**
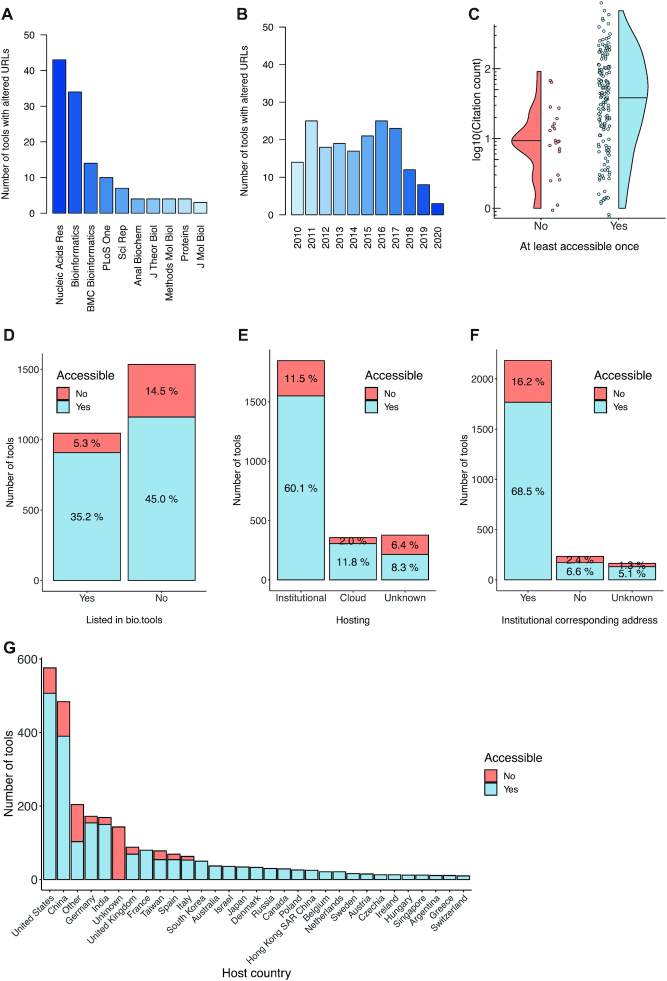
Distribution of publication metadata for altered (**A**–**C**) and all (**D**–**G**) tool URLs. (**A**) Bar chart for the number of tools with altered URL collected per journal. (**B**) Bar chart of the number of tools with altered URL by publication year. (**C**) Back-to-back violin-dot plot for the number of citations by accessibility status. (**D**) Stacked bar chart for the total number of tools and corresponding availability split by their presence in *bio.tools*. (**E**) Like in (D) but for determined host origins. (**F**) Like in (D) but for the type of corresponding e-mail address given in the associated publications. (**G**) Analogously to (D) but split by the web server host country. The special bars *Other* and *Unknown* summarize tools for countries with <10 web servers and indeterminable destination, respectively.

We also tracked which services modified their URL without providing a new link in a scientific publication, i.e. the 185 tools that were removed in our last filtering step (cf. Figure 1A & ‘Materials and Methods’ section). The top-ranking journals mirrored the larger distribution reported before (Figure 4A), however, the average publication year is notably shifted toward the early years considered in the study (Figure 4B). This matches our expectation for services to take several years before a new host URL is released. Nevertheless, we found that for those tools changing the URL offside the scientific literature, a higher citation was obtained on average when they were accessible at least once in our testing frame (Figure 4C), corroborating previous observations (16). We conclude that web server availability and community popularity are robust against sometimes inevitable URL modifications, an observation we largely attribute to the capabilities of modern search engines, which rapidly re-index new websites and their keywords in a few hours or days.

### Tool metadata sheds light onto global web server landscape

An intriguing question is whether publication or web server metadata can be used to judge the *a priori* likelihood of a tool to be inaccessible. Therefore, we collected various features for the total 2581 tools investigated (cf. Supplementary Tables S1 and 2). First, the community has built key resources such as *bio.tools* to index and track scientific web servers along their lifetime. Interestingly, 40.5% of the tools considered are contained in *bio.tools* and an overwhelming fraction were accessible (Figure 4D). As a matter of fact, the subset of tools not contained in the service comprises more non-reachable tools, both percentage- and count-wise. By analyzing the host services, we found 71.6% of the tools to be hosted by individual research institutions and another 13.8% managed by cloud services but with overall similar accessibility rates (Figure 4E). Likewise, the distribution of corresponding contact information highlighted most e-mail addresses to be institutional but when comparing fractions the instances with non-institutional addresses are more prone to be unavailable. (Figure 4F). In fact, institutional addresses can be affected by personnel relocation and thus become unavailable, while non-institutional addresses are less likely to change. Lastly, the distribution of host countries matches the global distribution of countries by Gross National Income with the United States, China, and Germany hosting the most scientific web servers, the latter of which is closely followed by India (Figure 4G). Remarkably, many European countries do not list a single web server instance that was inaccessible in our study.

### Analysis of impact reveals hallmarks of web server development

We next sought to investigate the relation between web server availability and number of citations for the respective manuscript, similar to the approach of Schultheiss *et al.* Comparing the web servers in our tool collection grouped by publication date, we asked whether tools published in 2010 only, 2010 to 2011, and 2010 to 2012 have a different, i.e. higher citation count on average if they were reachable at least once in our tracking frame, as opposed to those being not reachable at all. The resulting *P*-values of 1.278 × 10^−07^, 2.457 × 10^−11^ and 6.758 × 10^−15^, and the about five times higher mean citation counts for the first group in each of the comparisons, strongly support these hypotheses. However, we reason that simply guaranteeing a long-term availability does not necessarily pay off with a high citation count, as 70 tools, which were still available and published in between 2010 and 2012, had less than 10 citations. To the contrary, only four tools that had been published in this period were not reachable at all in our time frame, even though they all received more than 100 citations. Whether the causal implication is that tools being well-cited early following publication are also subject to better long-term maintenance, or the other way around, tools that are well maintained tend to be cited more often on the long run, remains to be shown, e.g. through invited host surveys. Nevertheless, besides the quality of the work and the breadth of scope, we propose that many other factors influence the long-term impact of a web server. For example, we found that tools providing only an IP address or which were hosted in user-home directories were overall considerably less reachable (only 31 out of 68 tools (45.6%) reachable over IP and 20 out of 37 tools (54.1%) hosted in home directories were still available).

Based on our and previous findings, we collected a set of guidelines split into four categories to delineate good web server development practices targeted for beginners in the field, all of which are easy to implement and ultimately can prevent major sustainability issues (Table 1). In general, the guidelines are designed to support *reproducibility* of computational results, *security* by enforcing service integrity and privacy of user data, the *maintainability* through environment isolation and dependency minimization and *usability* via complementary ways of access, e.g. through an API, or strict documentation policies. We also ordered the specific recommendations in each category by decreasing priority to simplify selection of the most important ‘DOs’ and ‘DO NOTs’. To enumerate on those, switching to production settings of all software components in-use, performing regular security updates, e.g. at least once every six months, and replacing standard admin access URLs and logins with hard to guess strings is essential for a reliable base level of security. To improve reproducibility, developers should encapsulate the software environment, e.g. through Docker, as much as possible, use proper code and data version control, e.g. using GIT, and publicly state any package dependencies and their version tested during development. For better maintainability, we recommend to minimize any effort that is needed to migrate the service, again by encapsulating the environment, properly fixing the software dependencies to prevent implicit updates when using package managers such as *conda* or *pip*, and document all required steps to reset the service, should it be necessary. Popular scripting languages like *Python* and *R* are especially vulnerable to implicit dependency updates as respective packages are updated at a high frequency. We also suggest developers to provide extensive sets of tutorials and example inputs or files to the user. Finally, hosting on official domain names instead of plain IP-addresses improves usability because names can be remembered and referred to significantly better than long numbers.

**Table 1.** Good practice guidelines for developing scientific web servers by category

Security	Reproducibility	Maintainability	Usability
Switch to production mode when deploying	Use virtual machines or container virtualization (e.g. Docker)	Minimize migration effort, encapsulate the software environment as much as possible	Create very detailed tutorials, one for each aspect of the web server
Perform regular security updates	Version control web server code and data	Keep all software dependencies fixed (e.g. YAML files)	Provide multiple example files
Do not use standard admin panel access domains and/or passwords	List software packages in use along with version numbers	Document internals as much as possible	Provide access over a domain name instead of an IP address
Escape user-input to prevent remote-code execution (e.g. SQL injection)	List main analysis parameters and provide timestamps in custom downloads (e.g. plots and tables)	Backup database onto external storage (e.g. user data)	Do not switch the top-level domain when publishing an update
Use DDoS protection service	Offer downloads for core data	Use popular frameworks, avoid implementing everything from scratch	Render progress bar and generate unique job ID for compute-intensive jobs
Use encryption (SSL/https)	Provide versioned subdomains and APIs	Avoid hosting in home directories, potentially depending on a user environment	Provide valid author contact details
Use valid SSL certificates to prevent malicious browser errors	Keep older versions running as archive	Include JavaScript libraries via CDN and keep a local copy as backup	Use a caching framework
Keep user submitted files private			Implement helper text messages
Set rate limits for public APIs			Use color-blind friendly palettes
Set file size limits for user uploads			Provide an (REST-ful) API in addition to standard interface
Set strict timeouts and use queue managers for compute intensive jobs			Announce maintenance slot to user before performing updates

Each column denotes a set of guidelines from the same category. Specific items in each category (column) are ordered by decreasing priority to simplify selection of the most important guidelines.

## DISCUSSION

With increasing frequency and broader applications, the importance of bioinformatics web services and web servers is growing. This calls for an in-depth consideration on the availability and sustainability of respective services, since it might have severe consequences for research projects. In case a web-based program is used in other manuscripts to present analyses and the original tool is discontinued, later publications can be impacted by non-reproducible results. Aims of our study were to present a comprehensive analysis of the availability of web services, to get insights into the dynamics and to monitor the availability over a longer period of time, and to get an understanding whether more recent web services can be rescued by contacting the corresponding authors. There are more measures that could be added to the analysis, e.g. usage rates, the number of update publications per tool, implementation technology and influence of international collaboration in the development of web servers. However, these aspects rather resemble a scientometric analysis (20), which does not belong to the core of our present study.

Among the most comprehensive articles on the availability of web based tools, Schultheiss *et al.* analyzed 927 web services published in the annual NAR Web Server Issues between 2003 and 2009 (15). Their test on the functionality on 77% of all tools showed that 13% were truly no longer working and for 45% of all services the functionality could be fully validated. A survey among 872 web server issue corresponding authors returned 274 replies, suggesting that the majority of tools are developed solely by students and researchers without a permanent position. Our analysis generalizes the results of the Schultheiss study. Around three times more tools were considered and also other journals than NAR were included. Additionally, we monitored the availability of web-based programs over a four-month period, which has not been performed in this manner before. Our results are nonetheless very well aligned with the observations by Schultheiss *et al.* described 10 years ago. We also provide a novel intervention experiment to demonstrate responsiveness and the estimated percentage of web servers that can be brought back to life by contacting corresponding authors.

It is important to elaborate on possible limitations of the present study. First, the literature search might already be biased since our search query requires the abstract to contain both, the keyword web service (or similar) and a web address and the strings ‘www’, ‘http’ or ‘https’. While this holds for many tools, obviously not all web servers are covered by a respective literature search leading to false negatives in our data set. A second limitation is the resolution of redirection triggers. While we followed html redirects in the download routine, other redirects were checked manually since they might also be triggered by client-side resolved JavaScript code. Whether and how redirects have been changed during the study runtime might also influence the results. A third limitation arises from the definition of availability. Many tools do not provide example files nor (RESTful-)APIs to test proper functionality in an automated fashion. In that, our analysis represents rather an upper boundary since a working main page of the web servers was already sufficient to count the tools as available. However, automatic testing the proper functionality for several thousand server instances without a common and standardized access interface is currently infeasible and requires extensive manual work. One strength of the study is at the same time a confounding factor: the rescue experiment potentially influenced the availability of tools. Likely, a substantial fraction of the 20 tools that were brought back to service by our e-mail initiative would have remained offline for a longer period of time without the intervention. Still, the 20 tools represent only a minor fraction of 0.8% of the 2581 tools included in the study. In the light of the on-going pandemic caused by SARS-CoV2, we did not detect a significant association between a reduced web server availability and the lockdown faced in most Asian and European countries between March and May of 2020. For three reasons this imaginable association is unlikely; First, our tracking frame reaches until the end of August, a time by which many universities returned to regular operations. Second, we compared availability for web servers hosted in Spain and Italy, the countries that were severely hit by the pandemic lockdown procedures and did not find an altered distribution of downtimes. Lastly, the reasons for server outage communicated by web server authors participating in the intervention experiment did not yield any COVID-19 related impact in all but one case. Similarly to the aforementioned analysis the common summer break, which is entirely contained in our tracking time-frame, did not have considerable influence on the availability rates, although it might be conceivable that the course of the summer break itself might have been altered by the SARS-CoV2 induced pandemic.

Our study raises questions about how to overcome the increasing trend of unavailable tools. First, cloud-based hosting and container-based applications such as Docker can simplify maintenance procedures and add to the reproducibility of research (21). In addition, open and comprehensive code-sharing is increasingly recognized and facilitated through major open-source platforms such as GitHub (https://github.com) and Docker hub (https://hub.docker.com). Further, recent community efforts such as *udocker* (22) promote usability of complex software tools by non-experts in multi-user environments, which closely matches most institutional compute server policies. Building upon these community efforts and our study results, we defined simple guidelines for developers that easily integrate into existing web server development workflows but are expected to substantially improve sustainability and long-term impact. Moreover, at best, one central repository would host a comprehensive list of web services. For this purpose several repositories and collections have already been established (e.g. https://www.biostars.org (23), https://bioinformaticssoftwareandtools.co.in/, or https://bio.tools/). Also, the EMBRACE Registry has been proposed as an active database for bioinformatics web services (24). Unfortunately, the web presence cannot be reached anymore (http://www.embraceregistry.net). Even though central and well-maintained databases are important, common standards and scientific guidelines become essential for large-scale data and code sharing practices. For example, ELIXIR (25) is one of the largest multi-national endeavors to integrate and coordinate computing facilities, web services, and databases across more than 220 research organizations. The FAIRsharing service (26) is a part of ELIXIR, providing community-based and reviewed standards/policies for sharing and maintaining databases. A comprehensive summary and detailed descriptions on the individual web service repositories can be found in (27).

Mechanisms for finding services automatically have already been discussed in 2008 (28) but still no perfect solution seems to exists and oftentimes manual curation is required. We suggest that a respective resource should contain at least the actual web link and a contact consisting of a full name and an e-mail address. Further, it would be desirable that web-based tools offer a well-defined API along with a standardized input file facilitating automated and daily remote tests. If testing fails, the respective contact can then be alerted automatically and mitigate the errors in due time. This could be a fair compromise to balance required efforts between the community, trying to keep the set of scientific web servers persistent, and the authors who need to provide suitable testing functionality on their services. It is conceivable for future artificial intelligence-based applications to further reduce manual intervention by automatically screening web sites to classify both availability and functionality. On the other hand, it is however also fair to mention that this task at present is implicitly performed on a large-scale by the entire research community.

As conclusion of our study we propose the timely development of a central web resource for monitoring the availability of web-based tools via automated API testing to generate on-going availability reports and statistics that serve both the web server developers and user community.

## DATA AVAILABILITY

All data are freely available as supplement to the manuscript.

## CODE AVAILABILITY

Computer code responsible to scan, download and aggregate web server statistics is available from https://github.com/CCB-SB/web-server-availability.

## Supplementary Material

gkaa1125_Supplemental_Files
