# Supplementary material for: On the lifetime of bioinformatics web services
Source: Nucleic Acids Res. 2020 Dec 3;48(22):12523–33. doi: 10.1093/nar/gkaa1125 (PMC7736811; doi:10.1093/nar/gkaa1125)
Supplement: gkaa1125_Supplemental_Files [file gkaa1125_supplemental_files.zip › Supplementary_materials-FK.docx]

**On the lifetime of bioinformatics web services**

**Supplementary Materials**

**Supplemental Table 1**: Compilation of curated web-tools with extracted primary and meta-data information used in the study.

**Supplemental Table 2**: Metadata and curated availability information for tools with altered publication URLs that were separated from the static and dynamic analysis.
